# Prevalence of Polycystic Ovarian Syndrome in India: A Systematic Review and Meta-Analysis

**DOI:** 10.7759/cureus.32351

**Published:** 2022-12-09

**Authors:** Mintu Dewri Bharali, Radhika Rajendran, Jayshree Goswami, Kusum Singal, Vinoth Rajendran

**Affiliations:** 1 Department of Community Medicine, Guwahati Medical College and Hospital, Guwahati, IND; 2 Department of Biotechnology, Indian Institute of Technology, Guwahati, Guwahati, IND; 3 Department of Obstetrics and Gynecology, Gauhati Medical College and Hospital, Guwahati, IND; 4 Center For Advanced Research Evidence-Based Child Health, Post Graduate Institute of Medical Education & Research (PGIMER), Chandigarh, IND; 5 Department of Community Medicine/Family Medicine, All India Institute of Medical Sciences, Jodhpur, IND

**Keywords:** indian women, meta-analysis, systematic review and meta-analysis, pcos, polycystic ovarian syndrome

## Abstract

Stein-Leventhal syndrome, often known as polycystic ovarian syndrome (PCOS), is a syndrome that affects women's reproductive health. PCOS is one of the most common endocrine and metabolic disorders in women of reproductive age. The etiology of PCOS remains unknown mainly, and the estimation of PCOS burden in a specific geographical location will impact disease control strategies. Hence, this study estimated the pooled prevalence of PCOS in Indian women. Databases such as PubMed, CINHAL, Scopus, and Google Scholar were thoroughly searched. Only those published Indian studies that reported the prevalence of PCOS from 2010 to 2021 and had at least one of the following diagnostic PCOS criteria were included in the systematic review: the National Institutes of Health (NIH), Rotterdam's criteria, or/and Androgen Excess Society (AES). MetaXL version 5.3 software was used for data analysis. The risk of bias was assessed using modified Joanna Briggs Institute criteria for cross-sectional studies. Out of 17132 articles, 11 articles were selected for systematic review and meta-analysis. The pooled prevalence of PCOS was 11.33(7.69-15.59) using the random effect. The proportion of Hirsute using the Ferriman-Gallwey score was highly variable, ranging from 1.6% to 37.9% (n=6). The prevalence rate of PCOS is high among Indian women. The pooled prevalence of PCOS was close to 10% using Rotterdam's criteria and AES criteria, while it was 5.8% using NIH criteria. The study's overall finding emphasizes the need for more acceptable and uniform diagnostic criteria for screening PCOS. At the same time, policy-makers should consider giving more importance to PCOS in their effort to control non-communicable diseases.

## Introduction and background

In the mid-1900s, Stein and Leventhal (Chicago, IL, USA) investigated the mechanisms of female sterility. According to Stein and Leventhal, women with sterility, equated with infertility, had abundant body hair and disturbed menstrual cycles. Irving Freiler Stein Sr. wrote "The Stein-Leventhal Syndrome: A Curable Form of Sterility" in 1958, detailing his findings on Stein-Leventhal syndrome diagnosis and surgical therapy. Stein-Leventhal syndrome, often known as polycystic ovarian syndrome (PCOS), is a syndrome that affects women's reproductive health. Excess hair in the body, absence of menstrual cycle (amenorrhea), and infertility are all common symptoms of PCOS [1]. In the 21st century, reproductive health remains a top public health priority issue that needs a holistic approach to address it.

PCOS is one of the most commonly reported endocrine and metabolic disorders among women of reproductive age. It is a heterogeneous condition characterized by features of androgen excess and ovarian dysfunction symptoms in the absence of another diagnosis. Although the etiopathology of PCOS is not so well proven, accumulating evidence suggests that it is a multi-gene condition with substantial epigenetic and environmental impacts, including nutrition and lifestyle variables. Menstrual abnormalities and reproductive dysfunction are the most commonly reported signs of PCOS, leading to female infertility [2,3]. Cardiovascular disease, hypertension, lipid metabolic problems, and endometrial cancer are all two to six times more common in PCOS patients than in the general population [4]. PCOS is easy to diagnose and treat; it just takes judicious utilization of already available standardized diagnostic tests and the application of appropriate approaches to address hyperandrogenism, the consequences of ovarian dysfunction, and the metabolic abnormalities that arise with it [5].

In the last few years, several attempts have been made to standardize the diagnostic criteria for PCOS [6]. But still, the diagnostic criteria for PCOS are debatable. First, in 1990, the National Institutes of Health (NIH) established criteria for PCOS [7], followed by Rotterdam criteria in 2003 [8]. This criterion involves the presence of any two of the three conditions: (a) oligomenorrhea/anovulation, (b) clinical/biochemical hyperandrogenism, and (c) polycystic ovaries (each ovary containing ≥12 follicles measuring 2-9 mm). In 2006, AES criteria were given by the Androgen Excess Society (AES), featuring clinical/biochemical hyperandrogenism with either oligo/anovulation or polycystic ovaries [9]. 

As indicated by the NIH diagnostic criteria, the revealed predominance of PCOS went from 6% to 9% in the United States, the United Kingdom, Spain, Greece, Australia, Asia, and Mexico [10]. Related to variances in research populations, limitations because of types of recruitment and sampling, and an absence of standardized definitions for the phenotypes, there is substantial disparity in reported prevalence even when using the same diagnostic criteria. The impact of race and nationality on the clinical presentation of androgen excess [11], as well as the gradual improvement in the presence of antral follicles by ultrasonography [12], may potentially impact the differences in reported prevalence. The ambiguity surrounding PCOS findings must be addressed promptly to give doctors and their patients more diagnostic accuracy, minimizing incorrect classification and the possible psychological distress that misdiagnosis can be caused by it [13].

The prevalence of a disease in a particular region is always a necessary tool for any control measures. However, there are no full-fledged published data on PCOS prevalence and distribution patterns in India because of an absence of well-designed studies with a robust methodology. As a result, a systematic review that provides a suitable pooled prevalence is highly required. With this goal, the present study was planned to measure the pooled prevalence of PCOS among Indian women from 2010 to 2021.

## Review

Methodology

This study was completed following the PRISMA (Preferred Reporting Items for Systematic Reviews and Meta-Analyses) checklist [14] and was registered in PROSPERO (CRD42021261617) [15]. The study framework was designed per the PRISMA guidelines before starting the literature search. No adjustments were made after that. The aim and objective of the study were to conduct a systematic review and meta-analysis to assess the pooled prevalence of PCOS in India from 2010 to 2021 using NIH, Rotterdam, and Androgen Excess (AE)-PCOS Society criteria.

Search method

Data sources such as PubMed, CINHAL, Scopus, and Google Scholar were systematically searched to find all the published studies reporting on the prevalence of PCOS in India till November 2021 by two blinded investigators (M.D.B. and V.R.). A complete electronic search strategy for each database was applied, and the search for published articles was thorough (Appendix Table 4).

Study selection

**Figure 1:**
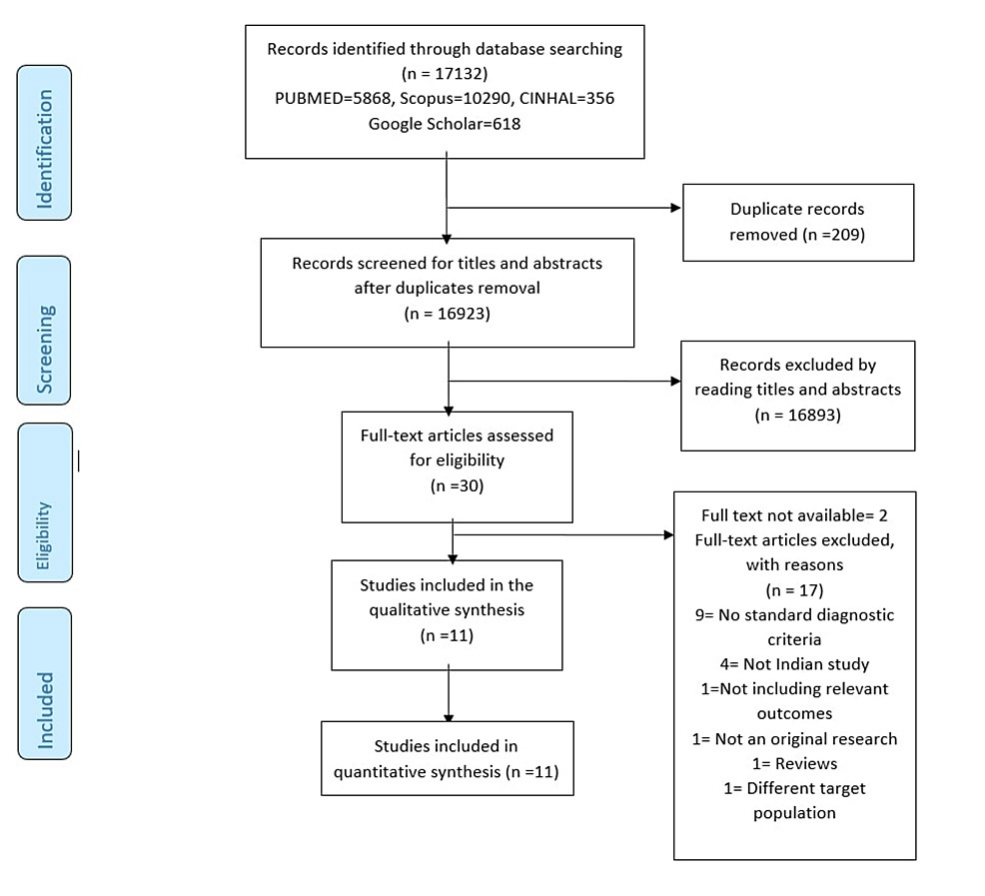
PRISMA flow diagram showing the study selection process

The eligibility criteria were pre-defined before conducting the literature search. Only those studies with criteria such as NIH, Rotterdam, or AE-PCOS used for the PCOS diagnosis were included in the systematic review [7-9]. The search was restricted to human studies, Indian studies with the English language, and publications from 2010 to 2021. If studies did not specify the diagnostic criteria applied, had no data regarding the prevalence, or were not published as peer-reviewed original research publications, they were eliminated. Two blinded investigators (M.D.B. and V.R.) conducted the initial searching and screening of titles and abstracts. After a full-text review regarding the inclusion of the particular study, the third investigator (JG) was consulted for the final decision. The initial search from PubMed, CINHAL, Scopus, and Google Scholar yielded a total of 17,132 articles (Figure 1). After the initial removal of duplicates, screening from abstracts and titles, only 30 relevant articles were undertaken for full-text review for eligibility. Furthermore, on the exclusion of 19 articles for various reasons (Figure 1), 11 articles were included in the quantitative synthesis.

Data extraction

Two authors (M.D.B. and V.R.) created a data table form for the data extraction process, which was pilot-tested to ensure author unanimity. Data extraction was done by all three Investigators (M.D.B., V.R., and J.G.) independently in pretested and piloted format in a Microsoft Excel sheet; regarding any disagreement on the extracted data, final consensus was made after discussion with the fourth investigator (R.R.). Data were extracted using Microsoft Excel sheet for the following variables: author, title, journal name, publication year, region, sampling frame, study setting, sample size, study population, risk-of-bias appraisal, and the criteria used to measure the prevalence of PCOS. The primary outcome was the pooled prevalence of PCOS.

Quality assessment of studies/risk of bias

The quality assessment of the included studies was done using the modified Joanna Briggs Institute (JBI) criteria by the University of Adelaide [16]. The bias risk was appraised by all three investigators by giving a response of "yes," "no," "unclear," and "not applicable." All three reviewers independently assessed the bias risk using the modified JBI criteria. In case of a mismatch of results, the common opinion of any two reviewers was the final decision. The evaluated articles were divided into three categories: high risk of bias (JBI score < 50%), moderate risk of bias (JBI score between 50% and 69%), and low risk of bias (JBI score ≥ 70%) [17].

Data analysis

MetaXL version 5.3 software was used for data analysis. Cochrane's Q test evaluated the probable sources of heterogeneity to identify the presence of heterogeneity, and I2 statistics were used to measure the amount of heterogeneity within and between studies using each of the three diagnostic criteria. Q test with p < 0.10 was considered statistically significant heterogeneity, and I2 > 75% was regarded as high heterogeneity [18]. The pooled prevalence of PCOS has been estimated using the random-effects model (DerSimonian-Laird method) [19]. Transformed double arcsine transformation has been used for stabilizing the variance of each study's proportions. Publication bias was evaluated using the Doi plot and Luis Furuya-Kanamori (LFK) asymmetry index [20]. Sensitivity analysis has been done to indicate the major determinant for the pooled prevalence of PCOS and to identify the main source of heterogeneity.

Results

A total of 30 articles were reviewed for a full text, and 11 articles were included in the present study [21-31]. Most of the selected studies were from Southern India, and none of the studies selected were from Eastern India. Table 1 shows the details of the selected study.

**Table 1:** Showing detailed characteristics of the included studies. AES, Androgen Excess Society; NIH, National Institutes of Health; PCOS, polycystic ovarian syndrome

Author, year	Region	Age group (years)	Criteria to diagnose PCOS	Sample size	Prevalence (%)
Nidhi et al., 2011 [21]	Andhra Pradesh	15-18	Rotterdam’s, NIH	460	9.13, 2.61
Vijaya and Bharatwaj, 2014 [22]	Pondicherry	19-25	Rotterdam’s	238	11.76
Bhuvanashree et al., 2013 [23]	Andhra Pradesh	10-19	Rotterdam’s	253	15.42
Joshi et al., 2014 [24]	Maharashtra	15-24	Rotterdam’s, AES	600	22.50, 10.67
Deswal et al., 2019 [25]	Haryana	16-45	Rotterdam’s	2253	4.17
Gupta et al., 2018 [26]	Madhya Pradesh	17-24	Rotterdam’s	500	8.20
Nanjaiah 2018 [27]	Karnataka	18-30	Rotterdam’s	396	4.55
Singh et al., 2018 [28]	Andhra Pradesh	15-19	Rotterdam’s	117	11.97
Laddad et al., 2019 [29]	Maharashtra	10-19	Rotterdam’s	150	17.33
Ganie et al., 2020 [30]	Kashmir	15-40	Rotterdam’s, NIH, AES	964	13.59, 11.11, 13.17
Kusuma et al., 2021 [31]	Telangana	15-45	Rotterdam’s	624	11.54

While performing the risk of bias assessment using modified JBI criteria (Table 2), most of the studies were based on community settings except for Singh et al.’s study [28] and Laddad et al’s study [29], which were carried out in the outpatient departments of hospitals. All the selected papers reported have used Rotterdam's criteria in addition to those three papers that used NIH and AES criteria. Most selected papers give details about oligo/amenorrhea except for one paper, Bhuvanashree et al. [23], where no detailed information was available for the study's diagnostic criteria.

**Table 2:** Risk of bias assessment of the included studies using the modified JBI criteria. JBI, Joanna Briggs Institute

Author	Nidhi et al. [21]	Vijaya and Bharatwaj [22]	Bhuvanashree et al. [23]	Joshi et al. [24]	Deswal et al. [25]	Gupta et al. [26]	Nanjaiah [27]	Singh et al. [28]	Laddad et al. [29]	Ganie et al. [30]	Kusuma et al. [31]
1. Was the sample frame appropriate to address the target population?	Yes	Yes	No	Yes	Yes	Yes	Yes	No	No	Yes	Yes
2. Were study participants sampled in an appropriate way?	No	Yes	No	Yes	Yes	Unclear	Yes	No	No	No	Yes
3. Was the sample size adequate?	Unclear	Unclear	Unclear	Yes	Yes	Yes	Yes	Unclear	Unclear	Unclear	Yes
4. Were the study subjects and the setting described in detail?	Yes	Yes	Yes	Yes	Yes	Yes	Yes	Yes	Yes	Yes	Yes
5. Was the data analysis conducted with sufficient coverage of the identified sample?	Yes	Yes	Yes	Yes	Yes	Yes	Yes	Yes	Yes	Yes	Yes
6. Were valid methods used for the identification of the condition?	Yes	Yes	Unclear	Yes	Yes	Unclear	Yes	Unclear	Yes	Yes	Yes
7. Was the condition measured in a standard, reliable way for all participants?	Yes	Yes	Unclear	Yes	Yes	Yes	Unclear	Unclear	Unclear	Yes	Yes
8. Was there appropriate statistical analysis?	Yes	Yes	Yes	Yes	Yes	Yes	Yes	Yes	Yes	Yes	Yes
9. Was the response rate adequate, and if not, was the low response rate managed appropriately?	Unclear	Yes	Yes	No	Yes	Yes	Yes	Yes	Yes	Unclear	Yes
Risk of bias	Moderate risk	Low risk	High risk	Low risk	Low risk	Low risk	Low risk	High risk	Moderate risk	Moderate risk	Low risk

Most studies defined oligomenorrhea as the menstrual cycle duration of more than 35/45 days or less than eight menses per year. The majority of the studies used the modified Ferriman-Gallwey criteria to diagnose clinical hyperandrogenism. The cut-off for most of the studies was eight, while only one study, Nidhi et al. [21], took six as the cut-off for diagnosing hirsutism. Out of the 11 studies, only six reported the prevalence of hirsute. Their frequency is highly variable, with the lowest being reported by Deswal et al. (1.6%) [25] and the highest by Ganie et al. (37.9%) [30]. Only four studies, Deswal et al. [25], Gupta et al. [26], Singh et al. [28], and Laddad et al. [29], reported other presentations of clinical hyperandrogenism such as acne, alopecia, and hyperpigmentation. Out of the 11 studies, only three studies, Nidhi et al. (2.8%) [21], Deswal et al. (2.7%) [25], and Kusuma et al. (12.3%) [31], reported the proportion of females presenting with biochemical hyperandrogenism, and most of them took more than two standard deviation of serum testosterone level in comparison to average women in their reproductive age groups as the cut-off for biochemical hyperandrogenism. Only five studies reported the prevalence of polycystic ovaries, and most of them took the total number of cysts per ovary (n>10-12) and ovarian volume > 10 ml as diagnostic criteria; in addition to, one study, Bhuvanashree et al. [23], also took bilateral presence of multiple sub-cortical ovarian cysts arranged in a necklace pattern as diagnostic criteria for polycystic ovaries.

Pooled prevalence of PCOS

**Figure 2:**
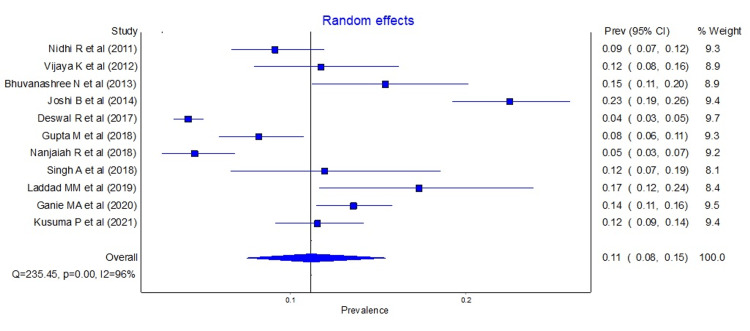
Forest plot showing the pooled prevalence of PCOS according to Rotterdam's criteria. PCOS, polycystic ovarian syndrome

All the selected studies reported the prevalence of PCOS using Rotterdam’s criteria, while only two studies (Nidhi et al. [21] and Ganie et al. [30]) used the NIH criteria prevalence and AES criteria (Joshi et al. [24] and Ganie et al. [30]). The prevalence of 11 studies using Rotterdam's criteria ranged from 4.2% to 22.5%. The pooled prevalence of eleven studies using Rotterdam's criteria was 11.33% (95% CI: 7.69 to 15.59), as shown in Figure 2.

**Figure 3:**
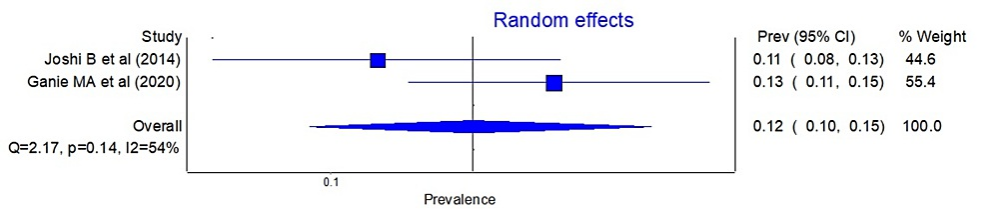
Forest plot showing the pooled prevalence of PCOS according to the AES criteria. AES, Androgen Excess Society

**Figure 4:**
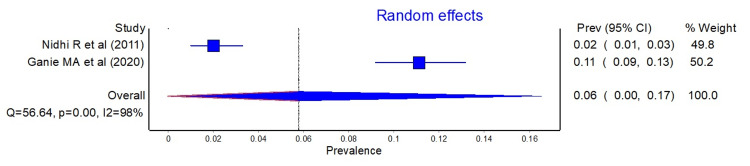
Forest plot showing the pooled prevalence of PCOS according to the NIH criteria. NIH, National Institutes of Health

Similarly, the pooled prevalence of PCOS using the AES and NIH criteria are shown in Figure 3 and Figure 4, respectively.

Heterogeneity and publication bias

**Figure 5:**
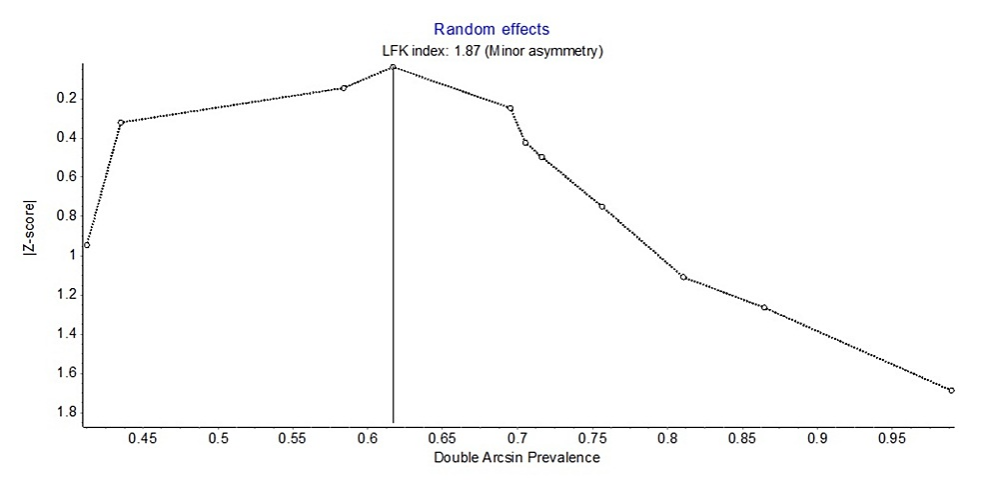
Showing publication bias using Doi plot.

The 11 included studies were analyzed for heterogeneity and publication bias. High heterogeneity was found in the analysis with the Q test (p <0.001) and I2 statistics (I2 = 96%). For publication bias, the Doi plot showed asymmetry confirming the presence of bias, and minor asymmetry was seen in the LFK index (LFK index = 1.87) (Figure 5).

Sensitivity analysis

Each study's effect (i.e. eleven studies) on the pooled prevalence of PCOS has been analyzed by excluding each study step by step using sensitivity analysis (Table 3). It showed that three studies (Joshi et al. [24], Deswal et al. [25], and Nanjaiah [27]) were comparatively the prime determinants of the pooled prevalence of PCOS, and the higher source of heterogeneity comes from the study by Nidhi et al. [21].

**Table 3:** Sensitivity analysis for the included studies according to Rotterdam's criteria

Excluded studies	Pooled prevalence (95% CI)	I^2^ (95% CI)	P-value
Nidhi et al., 2011 [21]	11.4 (7.3-16.2)	96.18 (94.50-97.34)	<0.001
Vijaya et al., 2014 [22]	11.1 (7.2-15.7)	96.14 (94.44-97.32)	<0.001
Bhuvanashree et al., 2013 [23]	10.8 (7.0-15.2)	96.00 (94.22-97.24)	<0.001
Joshi et al., 2014 [24]	10.2 (7.1-13.8)	93.66 (90.30-95.85)	<0.001
Deswal et al., 2019 [25]	12.2 (9.0-15.7)	90.71 (85.06-94.23)	<0.001
Gupta et al., 2018 [26]	11.5 (7.4-16.3)	96.17 (94.49-97.34)	<0.001
Nanjaiah [27]	12.0 (8.0-16.7)	95.95 (94.13-97.20)	<0.001
Singh et al., 2018 [28]	11.1 (7.2-15.6)	96.16 (94.47-97.33)	<0.001
Laddad et al., 2019 [29]	10.7 (6.9-15.0)	96.01 (94.24-97.24)	<0.001
Ganie et al., 2020 [30]	10.9 (7.0-15.6)	95.76 (93.83-97.09)	<0.001
Kusuma et al., 2021 [31]	11.1(7.1-15.9)	96.10 (94.38-97.30)	<0.001

Discussion

PCOS is the most commonly reported endocrine issue in women of reproductive age. Its clinical presentations are quite diverse, making it easy to confuse it with other endocrine diseases such as hyperprolactinemia, abnormal thyroid function, and congenital adrenal hyperplasia [32,33]. The current paper is the first systematic review and meta-analysis to estimate the overall prevalence of PCOS in India as per three diagnostic criteria. This paper demonstrates that the pooled PCOS prevalence estimates according to Rotterdam’s criteria is 11.34% in India. These findings are slightly higher when compared to the meta-analysis conducted by Wu et al., where the overall prevalence of PCOS was 10.01% among Chinese women [34]. The PCOS prevalence rates among Chinese females varied by region; the prevalence rates of PCOS in eastern regions (7.82%) are much lower than those in central (14.24%) and western regions (13.35%) [34]. Since only a few published articles were found in India on the prevalence of PCOS, we could not perform a subgroup analysis based on regions in India. The prevalence of PCOS diagnosed using Rotterdam’s criteria (2003) is reportedly higher than the NIH criteria (1990) and AES Criteria (2006) [35]. According to a few studies, Rotterdam’s criteria may include some individuals with mild phenotypes of PCOS in the diagnosis, thereby raising PCOS prevalence [36].

The prevalence of PCOS had significant differences among regions, occupation, age, time of publication, diagnostic criteria, and survey populations [34]. The current systematic review shows a difference in the PCOS diagnostic criteria across the included studies. Regarding hyperandrogenism, the cut-off score of modified Ferriman-Gallwey criteria used for hirsutism and the biochemical parameters measuring hormones for hyperandrogenemia was uneven throughout the studies. For example, Nidhi et al.'s study stated that they reported prevalence according to Rotterdam’s criteria, which included women with an F-G score cut-off of ≥6 to diagnose hirsutism [21]. However, five studies have stated that they have used an F-G score cut-off of ≥8 to classify it as hirsutism [24,28-31]. Similarly, Skiba et al.’s study found a lack of adherence to the recognized PCOS diagnostic criteria across various studies. It further stated that consistent use of Rotterdam’s criteria in the research context is complex, and it might raise further issues about its utility as a diagnostic framework [13]. 

Since the threshold used to measure PCOS by ultrasonography is not mentioned in all the studies included in the current systematic review, it could have led to discrepancies among studies. However, it is unclear if the variation in PCOS prevalence is linked to different thresholds used for measuring the antral follicle count (AFC) and ovarian volume, necessitating more study in this area. The frequency of the transducer used to define PCOs morphology may also have a role in the disparities in prevalence rates [6,37,38].

Including school and college-going adolescents in this current review may have inflated the pooled prevalence estimate. Similarly, Joshi et al.'s study included adolescents and young girls in Mumbai, revealing the highest prevalence of PCOS estimates using Rotterdam’s criteria [24]. Various studies had stated that the inclusion of adolescents in their samples might amplify the prevalence estimate when Rotterdam’s criteria were used, as both oligo-anovulation and PCOS are common in adolescent girls [39,40]. Furthermore, students have long mental work hours and may be under long-term stress, resulting in increased catecholamine secretion, endocrine function disorder, sympathetic nerve excitability, and secretion of hypothalamus-pituitary-adrenal cortex hormone, all of which reduce immune function. Students frequently make poor lifestyle choices, such as inconsistent eating and little exercise. These variables might hasten the onset of PCOS [34,41].

Though the quality of the study was appraised using the JBI criteria, this paper failed to assess the standard of individual diagnostic methods used to evaluate each diagnostic criterion for PCOS. Furthermore, the age group of all the studies is not uniform. Regional variations were not found as most of the studies are from the southern region of India, and none were from the Eastern part of India; therefore, the result may not reflect India as a whole.

Data availability statement

Data available within the article or its supplementary materials (Appendix Table 5).

## Conclusions

The pooled prevalence of PCOS was close to 10% using Rotterdam's criteria and AES criteria, while it was 5.8% using the NIH criteria. The study's overall finding emphasizes the need for more acceptable and uniform diagnostic criteria for screening PCOS. Although physicians are crucial in identifying PCOS and educating the public about this condition, the extra cost and amount of time it takes for a diagnosis and treatment may deter some young women from seeking assistance. Additionally, it is critical for healthcare professionals to communicate this information with cultural sensitivity. The guidelines for the management and awareness of PCOS in India need to be established with the assistance of this evidence by policy-makers, government organizations, and healthcare professionals.

## Appendices

**Table 4:** Search strategy from databases: PubMed, CINHAL, Scopus, and Google Scholar.

PubMed- 5868 (("polycystic ovary syndrome"[MeSH Major Topic] AND ("epidemiology"[MeSH Subheading] OR "epidemiology"[All Fields] OR "prevalence"[All Fields] OR "prevalence"[MeSH Terms] OR "prevalance"[All Fields] OR "prevalences"[All Fields] OR "prevalence s"[All Fields] OR "prevalent"[All Fields] OR "prevalently"[All Fields] OR "prevalents"[All Fields])) OR ("polycystic ovary syndrome"[MeSH Major Topic] AND ("cross sectional studies"[MeSH Terms] OR ("cross sectional"[All Fields] AND "studies"[All Fields]) OR "cross sectional studies"[All Fields] OR ("cross"[All Fields] AND "sectional"[All Fields]) OR "cross sectional"[All Fields])) OR ("polycystic ovary syndrome"[MeSH Major Topic] NOT "review"[Title/Abstract])) AND (2010:2021[pdat]) Filter: the English Language, Humans
Scopus- 10290 TITLE-ABS-KEY ( "polycystic ovary syndrome" ) 22330 ( TITLE-ABS-KEY ( "polycystic ovary syndrome" ) OR TITLE-ABS-KEY ( "polycystic ovarian syndrome" ) ) 24422 ( TITLE-ABS-KEY ( "polycystic ovary syndrome" ) OR TITLE-ABS-KEY ( "polycystic ovarian syndrome" ) OR TITLE-ABS-KEY ( "stein leventhal syndrome" ) ) 24712 ( TITLE-ABS-KEY ( "Polycystic ovary syndrome" ) OR TITLE-ABS-KEY ( "Polycystic ovarian syndrome" ) OR TITLE-ABS-KEY ( "Stein Leventhal Syndrome" ) OR TITLE-ABS-KEY ( "sclerocystic ovary syndrome" ) ) 24717 ( TITLE-ABS-KEY ( "Polycystic ovary syndrome" ) OR TITLE-ABS-KEY ( "Polycystic ovarian syndrome" ) OR TITLE-ABS-KEY ( "Stein Leventhal Syndrome" ) OR TITLE-ABS-KEY ( "sclerocystic ovary syndrome" ) OR TITLE-ABS-KEY ( "sclerocystic ovarian degeneration" ) ) 24718 ( TITLE-ABS-KEY ( "Polycystic ovary syndrome" ) OR TITLE-ABS-KEY ( "Polycystic ovarian syndrome" ) OR TITLE-ABS-KEY ( "Stein Leventhal Syndrome" ) OR TITLE-ABS-KEY ( "sclerocystic ovary syndrome" ) OR TITLE-ABS-KEY ( "sclerocystic ovarian degeneration" ) ) 24718 ( TITLE-ABS-KEY ( "Polycystic ovary syndrome" ) OR TITLE-ABS-KEY ( "Polycystic ovarian syndrome" ) OR TITLE-ABS-KEY ( "Stein Leventhal Syndrome" ) OR TITLE-ABS-KEY ( "sclerocystic ovary syndrome" ) OR TITLE-ABS-KEY ( "sclerocystic ovarian degeneration" ) OR TITLE-ABS-KEY ( "sclerocystic ovaries" ) ) 24742 ( TITLE-ABS-KEY ( "Polycystic ovary syndrome" ) OR TITLE-ABS-KEY ( "Polycystic ovarian syndrome" ) OR TITLE-ABS-KEY ( "Stein Leventhal Syndrome" ) OR TITLE-ABS-KEY ( "sclerocystic ovary syndrome" ) OR TITLE-ABS-KEY ( "sclerocystic ovarian degeneration" ) OR TITLE-ABS-KEY ( "sclerocystic ovaries" ) ) AND ( LIMIT-TO ( PUBYEAR , 2021 ) OR LIMIT-TO ( PUBYEAR , 2020 ) OR LIMIT-TO ( PUBYEAR , 2019 ) OR LIMIT-TO ( PUBYEAR , 2018 ) OR LIMIT-TO ( PUBYEAR , 2017 ) OR LIMIT-TO ( PUBYEAR , 2016 ) OR LIMIT-TO ( PUBYEAR , 2015 ) OR LIMIT-TO ( PUBYEAR , 2014 ) OR LIMIT-TO ( PUBYEAR , 2013 ) OR LIMIT-TO ( PUBYEAR , 2012 ) OR LIMIT-TO ( PUBYEAR , 2011 ) OR LIMIT-TO ( PUBYEAR , 2010 ) ) 14095 ( TITLE-ABS-KEY ( "Polycystic ovary syndrome" ) OR TITLE-ABS-KEY ( "Polycystic ovarian syndrome" ) OR TITLE-ABS-KEY ( "Stein Leventhal Syndrome" ) OR TITLE-ABS-KEY ( "sclerocystic ovary syndrome" ) OR TITLE-ABS-KEY ( "sclerocystic ovarian degeneration" ) OR TITLE-ABS-KEY ( "sclerocystic ovaries" ) ) AND ( LIMIT-TO ( PUBYEAR , 2021 ) OR LIMIT-TO ( PUBYEAR , 2020 ) OR LIMIT-TO ( PUBYEAR , 2019 ) OR LIMIT-TO ( PUBYEAR , 2018 ) OR LIMIT-TO ( PUBYEAR , 2017 ) OR LIMIT-TO ( PUBYEAR , 2016 ) OR LIMIT-TO ( PUBYEAR , 2015 ) OR LIMIT-TO ( PUBYEAR , 2014 ) OR LIMIT-TO ( PUBYEAR , 2013 ) OR LIMIT-TO ( PUBYEAR , 2012 ) OR LIMIT-TO ( PUBYEAR , 2011 ) OR LIMIT-TO ( PUBYEAR , 2010 ) ) AND ( LIMIT-TO ( DOCTYPE , "ar" ) ) 10290
CINHAL- 356 Search ID# Search Terms Search Options Actions S10 S8 AND S9 Limiters - Full Text Expanders - Also search within the full text of the articles; Apply equivalent subjects Search modes - Boolean/Phrase 356 S9 cross sectional study Limiters - Full Text Expanders - Also search within the full text of the articles; Apply equivalent subjects Search modes - Boolean/Phrase 91,516 S8 S6 AND S7 Limiters - Full Text Expanders - Also search within the full text of the articles; Apply equivalent subjects Search modes - Boolean/Phrase 1,716 S7 Prevalence Limiters - Full Text Expanders - Also search within the full text of the articles; Apply equivalent subjects Search modes - Boolean/Phrase 286,141 S6 S1 OR S2 OR S3 OR S4 OR S5 Limiters - Full Text Expanders - Also search within the full text of the articles; Apply equivalent subjects Search modes - Boolean/Phrase 4,608 S5 Sclerocystic Ovary Syndrome Limiters - Full Text Expanders - Also search within the full text of the articles; Apply equivalent subjects Search modes - Boolean/Phrase 327 S4 PCOS Limiters - Full Text Expanders - Also search within the full text of the articles; Apply equivalent subjects Search modes - Boolean/Phrase 3,115 S3 Stein Leventhal Syndrome Limiters - Full Text Expanders - Also search within the full text of the articles; Apply equivalent subjects Search modes - Boolean/Phrase 344 S2 Polycystic Ovarian Syndrome Limiters - Full Text Expanders - Also search within the full text of the articles; Apply equivalent subjects Search modes - Boolean/Phrase 1,185 S1 Polycystic Ovary Syndrome Limiters - Full Text Expanders - Also search within the full text of the articles; Apply equivalent subjects Search modes - Boolean/Phrase 2,078
Google Scholar- 618 allintitle: prevalence OR cross OR sectional OR pcos OR pcod OR pcod "polycystic ovarian syndrome" OR "polycystic ovarian disease"

**Table 5:** Data extraction for all the included studies. a/b, found/examined; AES, Androgen Excess Society; AFC, antral follicle count; DHEAS, dehidroepiandrostenedione sulphate; FAI, free androgen index; fT, free testosterone; HA, hyperandrogenemia; HS, hirsutism; mFG, modified Ferriman-Gallwey scoring; MH, based on menstrual history; NA, not available or not applicable; NIH, National Institutes of Health; OA, oligoanovulation; OV, ovarian volume; P, based on progesterone level; PCO, polycystic ovary; SHbg, sex hormone binding globulin; TT, total testosterone

S.no	Title	Author	Journal	Year	Design	Sampling frame size	Age group	Region	1. Was the sample frame appropriate to address the target population?	2. Were study participants sampled in an appropriate way?	3. Was the sample size adequate?	4. Were the study subjects and the setting described in detail?	5. Was the data analysis conducted with sufficient coverage of the identified sample?	6. Were valid methods used for the identification of the condition?	7. Was the condition measured in a standard, reliable way for all participants?	8. Was there appropriate statistical analysis?	9. Was the response rate adequate, and if not, was the low response rate managed appropriately?	Sample size	Error	Sample size calculated	Effective sample size	Non-response rate(assumed)	Effective non response rate)	NIH(no of case)	Rotterdam(no of case)	AES (no of case)	HS, mF-G, [HS a/b, (≥mF−G)]	mF-G	HA a/b, (biomarkers)	TT, fT, androstenedione, DHEAS, FAI	Acne	Hair loss/alopecia	Hyperpigmentation	OA a/b, (diagnosis)	MH, P, LH/FSH≥1	PCO on USG [PCO a/b, (AFC, OV)]	AFC, OV	Study population	Eligibility criteria	Management of hormonal use
1	Prevalence of Polycystic Ovarian Syndrome in Indian Adolescents	Nidhi et al.	J pediatric adolesc gynecol	2011	Cross-sectional	460	15-18	Andhra Pradesh	Yes	No	Unclear	Yes	Yes	Yes	Yes	Yes	Unclear	50	5	500	460	Na	11.5	12	42	NA	NA	Clinical hyperandrogenism: mFG score of 6 or higher	13/460	13/460, NA, not clear, clinical hyperandrogenism, biochemical hyperandrogenism: serum testosterone level of >82 ng/dL in the absence of other causes of hyperandrogenism.	NA	NA	NA	59/460	59/460, MH- oligo/amenorrhea: absence of menstruation for 45 days or more and/or ≤8 menses per year	42/55	42/55, Presence of >10 cysts, 2 to 8 mm in diameter, usually combined with increased ovarian volume of >10 cm^3^, and an echo-dense stroma in pelvic ultrasound scan.	Adolescent girls aged 15 to 18 years from a College in Anantapur, Andhra Pradesh	All participants were clinically healthy and none were suffering from chronic or acute diseases.	Use of oral contraceptive pills mentioned
2	Prevalence and Undetected Burden of Polycystic Ovarian Syndrome (PCOS) Among Female Medical Undergraduate Students in South India—A Prospective Study in Pondicherry	Vijaya and Bharatwaj	Global Journal for Research Analysis	2014	Cross-sectional	259	19-25	Pondicherry	Yes	Yes	Unclear	Yes	Yes	Yes	Yes	Yes	Yes	259	-	259	238	Na	8	NA	28	NA	17/28	17/28, HS measured by mFG score ≥8, with or without acne and/or androgenic alopecia.	NA	Biochemical hyperandrogenism was considered present with a serum testosterone level 2 standard deviations above the mean of normal women of reproductive age in the absence of other causes of hyperandrogenism	NA	NA	NA	27/28	27/28, MH: oligomenorrhea (Absence of menstruation for ≥35 days at least four times a year) or amenorrhea (No menstruation continuously for 6 months)	NA	Polycystic ovaries on ultrasound scanning was defined as an ovarian volume greater than 10 cm^3^ and/or 10 or more 2 to 8 mm follicles in a single plane when ultrasonography was performed	Female medical undergraduate students at Sri Lakshmynarayana Institute of Medical Sciences, Pondicherry (SLIMS)	Who gave informed consent	Not reported
3	Polycystic Ovarian Syndrome: Prevalence and Its Correlates Among Adolescent Girls	Bhuvanashree et al	Annals of Tropical Medicine & Public Health	2013	Cross-sectional	253	10-19	Andhra Pradesh	No	No	Unclear	Yes	Yes	Unclear	Unclear	Yes	Yes	253`		300	253	-	15.6	NA	39	NA	NA	FG scale	NA	NA	NA	NA	NA	NA	NA	NA	Bilateral presence of multiple subcortical ovarian cysts arranged in a necklace pattern	The subjects were selected randomly from the educational institutions such as schools, colleges (nursing, medical, junior colleges, college of yoga and naturopathy) and girl’s hostels of Nellore, in Andhra Pradesh.	In case an individual being lesser than 18 years, consent was obtained from the parent/guardian. Girls who have not attained menarche were excluded.	Not reported
4	A Cross-Sectional Study of Polycystic Ovarian Syndrome Among Adolescent and Young Girls in Mumbai, India	Joshi et al	Indian journal of endocrinology and metabolism	2014	Cross-sectional	778	15-24	Maharastra	Yes	Yes	Yes	Yes	Yes	Yes	Yes	Yes	No	10	2	1000	600	10	13.5	NA	135	64	82/600	82/600, ≥8 mFG	NA	FAI level of two standard deviations above the mean level among normal controls (formula: TT/SHbg × 100).	NA	NA	NA	148/600	148/600, MH: irregular cycles, amenorrhoea ( absence of periods for at least 3 of the previous cycle intervals or 6 months of amenorrhea) or oligomenorrhea (infrequent menstruation for ≥35 days)	229/600	229/600, AFC, OV, 12 or more follicles measuring 2-9 mm in diameter with or without ovarian volume >10 mL	Female population in the age group of 15-24 years	Girls aged 15-24 years, who had attained menarche more than 2 years before the study, who were unmarried and were willing to participate in the study were enrolled	Not reported
5	Cross-Sectional Study of the Prevalence of Polycystic Ovary Syndrome in Rural and Urban Populations	Deswal et al	International Journal of Gynecology & Obstetrics	2019	Cross-sectional	2400	16-45	Haryana	Yes	Yes	Yes	Yes	Yes	Yes	Yes	Yes	Yes	2400	20	2400	2253	20	9.3	NA	94	NA	28/94	28/94, Clinical hyperandrogenism- HS	61/94	61/94, Biochemical hyperandrogenism- elevated testosterone	38/94	14/94	NA	73-21/94	MI: oligomenorrhea 73/94, MI: amenorrhea 21/94	33/94	33/94, PCOS	Women of reproductive age (16–45 years) in Haryana	Women of reproductive age (16–45 years) who had irregular periods and clinical or biochemical hyperandrogenism (HS or elevated testosterone) and/or PCOS on ultrasound (Rotterdam criteria) were included in the study of PCOS. Women with thyroid abnormalities, adrenal hyperplasia, prolactin excess, or Cushing syndrome, all of which mimic the symptoms of PCOS, were excluded.	Considered- Not taking medications or oral contraceptive pills in the past 6 months.
6	A Cross Sectional Study of Polycystic Ovarian Syndrome Among Young Women in Bhopal, Central India	Gupta et al	International journal community medicine & public health	2018	Cross-sectional	500	17-24	Madhya pradesh	Yes	Unclear	Yes	Yes	Yes	Unclear	Yes	Yes	Yes	385	3	500	500	20	0	NA	41	NA	8/500	8/500, NA	NA	HS score of more than 8 was considered positive for HA.	409/500	103/500	NA	19/500	19/500, MH: oligomenorrhea after two years of menarche or primary amenorrhea at the age of 16 years	12/500	12/500, polycystic ovaries on ultrasound along with ovarian size of more than 10 cm	Girls of age group of 17-24 years studying girl’s colleges in different quadrants of Bhopal city in Madhya Pradesh, India	Girls aged 17-24 years, who had attained menarche more than 2 years before the study were enrolled. Exclusion criteria: those who were known cases of thyroid disorders, those with Cushing's syndrome, and who were not willing to participate were excluded from this study.	Not reported
7	Prevalence of Polycystic Ovarian Syndrome Among Female Students: A Cross-Sectional Study	Nanjaiah	National Journal of Community Medicine	2018	Cross-sectional	405	18-30	Karnataka	Yes	Yes	Yes	Yes	Yes	Yes	Unclear	Yes	Yes	405	20	405	396	15	-	NA	18	NA	65/396	65/396, NA	NA	NA, hyperandrogenism (clinical or biochemical)	NA	NA	92/396	60/396	60/396, NA, MH: menstrual irregularity, 24/396, menorrhagia/oligomenorrhea	NA	NA, Presence of 12 or more follicles in each ovary measuring 2–9 mm in diameter, and/or increased ovarian volume ( 10 mL)	Female students pursuing degrees at Maharanis College of science and Maharanis College of Arts & Commerce at Mysore.	Girls aged 18 to 35 years who have attained menarche were included. Girls who attained menarche in the past two years and who are suffering from congenital adrenal hyperplasia, androgen-secreting tumors, thyroid disorders, and hyperprolactinemia were excluded.	Not reported
8	Prevalence of Polycystic Ovarian Syndrome Among Adolescent Girls: A Prospective Study	Singh et al.	International Journal of Reproduction, Contraception, Obstetrics and Gynecology	2018	Cross-sectional	117	15-19	Andhra Pradesh	No	No	Unclear	Yes	Yes	Unclear	Unclear	Yes	Yes	117	Na	117	117	Na	0	NA	14	NA	3/117	Clinical hyperandrogenism: mFG score of 8 or higher	NA	NA, DHEA-sulfate, androstenedione.	9/117	1/117	5/117	22/117	22/117, MH: oligo/amenorrhea: absence of menstruation for 45 days or more and/or less than 8 menses per year.	NA	Presence of more than 10 cysts, 2-8 mm in diameter, usually combined with increased ovarian volume of more than 10 cm^3^, and an echo-dense stroma in pelvic ultrasound scan.	Adolescent girls aged 15 to 19 years attending OPD at the Department of Obstetrics and Gynaecology; Government Maternity Hospital, Sultan Bazaar, Osmania Medical College, Hyderabad.	Adolescents aged 15–19 years, not married, and had menarche more than 2 years before the study were included. Those who were known cases of thyroid disorders, hyperprolactinemia, Cushing's syndrome, and who were not willing to participate, were excluded.	Not reported
9	Study of Prevalence and Determinants of Polycystic Ovarian Syndrome Among Adolescent Girls in Rural Area: A Prospective Study	Laddad et al.	International Journal of Reproduction, Contraception, Obstetrics and Gynecology	2019	Cross-sectional	150	10-19	Maharastra	No	No	Unclear	Yes	Yes	Yes	Unclear	Yes	Yes	150	Na	150	150	Na	0	NA	26	NA	5/150	Clinical hyperandrogenism: mFG score of 8 or higher	NA	Persistent elevation of serum total and/or free testosterone.	16/150	2/150	8/150	40/150	MH: oligo/amenorrhea: Absence of menstruation for 45 days or more and/or less than 8 menses per year. Evidence of ovulatory dysfunction included consecutive menstrual intervals of more than 90 days, 1 year after menstrual onset; menstrual intervals persistently less than 21 days or more than 45 days 2 or more years after menarche.	NA	Polycystic ovaries: Presence of more than 12 follicles, 2-9 mm in diameter arranged peripherally, usually combined with increased ovarian volume of more than 10 cm^3^, and an echo-dense stroma in pelvic ultrasound scan	Adolescent girls aged 10 to 19 years attending OPD at the Department of Obstetrics and Gynaecology; Krishna Institute of Medical Sciences, Deemed University, Karad, Maharashtra, India.	Inclusion criteria- Adolescent girls in the age group of 10-19 years, adolescent girls had attained menarche, adolescents girls who have come to seek treatment from obstetrics and gynecology and adolescent OPD of Krishna Hospital. Exclusion criteria: known cases of thyroid disorders, hyperprolactinemia, Cushing's syndrome, those not willing to participate, married, and pregnant adolescents.	Not reported
10	Prevalence of Polycystic Ovary Syndrome (PCOS) Among Reproductive Age Women From Kashmir Valley: A Cross-Sectional Study	Ganie et al.	International Journal of Gynecology & Obstetrics	2020	Cross-sectional	3300	15-40	Kashmir	Yes	No	Unclear	Yes	Yes	Yes	Yes	Yes	Unclear	964	-	964	964	Na	45	107	131	127	366/964	mFG score of 8 or higher	NA	HA was defined as serum TT over 65 ng/dL, which represents the 97th percentile in a previous study of healthy women	NA	NA	NA	252/964	252/964- menstrual irregularity. Oligomenorrhea was defined as occurrence of eight or fewer menses per year or an intermenstrual interval of less than 35 days.	NA	Presence of 12 or more follicles of 2–9 mm in diameter and/or an ovarian volume higher than 10 mL in one or both ovaries.	Women in various educational institutes across the Kashmir valley.	Those who gave consent	Not reported
11	A Cross Sectional Study on, Prevalence of Polycystic Ovarian Syndrome and Its Health Effects, in Reproductive Age Women (15-45 Years) in a Rural Area, Telangana, India	Kusuma et al.	Int J Clin Obstet Gynaecol	2021	Cross-sectional	660	15-45	Telanagana	Yes	Yes	Yes	Yes	Yes	Yes	Yes	Yes	Yes	660	2	660	624	15	9.4	NA	72	NA	NA	172/624, clinical hyperandrogenism was defined as the presence of acne, alopecia, and HS recorded as mFG score ≥ 8.	77/182- Biochemical hyperandrogenism	Biochemical hyperandrogenism: 70/182; fT: 68/182; Androstenedione: 68/182; biochemical hyperandrogenism was defined as elevated free testosterone levels (>0.034 nmol/L) or raised FAI (formula: TT/SHbg × 100).	NA	NA	NA	153/624	MH: OA was defined as a cycle length 35 days or amenorrhea	67/182	67/182, polycystic ovaries was defined as ≥15 follicles measuring 2– 10 mm in diameter or ovarian volume 10 ml in at least one ovary	Reproductive age women (15-45 years of age) in a rural area of Telangana	Inclusion criteria: women in the 15- to 45-year age group previously diagnosed with or without PCOS who gave informed consent to participate in the study. Exclusion criteria: menarche within the past 2 years, post-menopausal women, pregnant and lactating women, women on oral contraceptive pills and intrauterine devices, those diagnosed with cancers, and women who underwent hysterectomy or bilateral oophorectomy.	Not reported
